# Current and Stray Flux Combined Analysis for Sparking Detection in DC Motors/Generators Using Shannon Entropy

**DOI:** 10.3390/e26090744

**Published:** 2024-08-30

**Authors:** Jorge E. Salas-Robles, Vicente Biot-Monterde, Jose A. Antonino-Daviu

**Affiliations:** 1 Escuela Técnica Superior de Ingeniera Aeroespacial y Diseño Industrial, Universitat Politècnica de València (UPV), Camino de Vera s/n, 46022 Valencia, Spain; jesalas@etsid.upv.es; 2 Instituto Tecnológico de la Energía, Universitat Politècnica de València (UPV), Camino de Vera s/n, 46022 Valencia, Spain; vibiomon@die.upv.es

**Keywords:** current signals, stray flux signals, Shannon entropy, brushed DC machines

## Abstract

Brushed DC motors and generators (DCMs) are extensively used in various industrial applications, including the automotive industry, where they are critical for electric vehicles (EVs) due to their high torque, power, and efficiency. Despite their advantages, DCMs are prone to premature failure due to sparking between brushes and commutators, which can lead to significant economic losses. This study proposes two approaches for determining the temporal and frequency evolution of Shannon entropy in armature current and stray flux signals. One approach indirectly achieves this through prior analysis using the Short-Time Fourier Transform (STFT), while the other applies the Stockwell Transform (S-Transform) directly. Experimental results show that increased sparking activity generates significant low-frequency harmonics, which are more pronounced compared to mid and high-frequency ranges, leading to a substantial rise in system entropy. This finding enables the introduction of fault-severity indicators or Key Performance Indicators (KPIs) that relate the current condition of commutation quality to a baseline established under healthy conditions. The proposed technique can be used as a predictive maintenance tool to detect and assess sparking phenomena in DCMs, providing early warnings of component failure and performance degradation, thereby enhancing the reliability and availability of these machines.

## 1. Introduction

Brushed DC motors and generators (DCMs) are crucial in various applications, especially in the automotive industry for electric vehicles (EVs), plug-in hybrid electric vehicles (PHEVs), and battery electric vehicles (BEVs) due to their high torque, power, and efficiency [1]. They are also employed in process industries such as metallurgy, paper and pulp, oil and gas, food and beverage, mining, and chemical industries, as well as in power generation. Despite their advantages, DCMs are susceptible to premature failure due to sparking between brushes and commutators. This issue can lead to unplanned outages and significant economic losses, highlighting the need for effective diagnostic techniques to enhance their reliability and availability [2].

The commutator/brush system is a critical failure point in DCMs, often due to factors such as excessive and uneven brush wear, defective contacts from improper spring tension, vibrations, contamination deposits on the commutator surface, and commutator deformation [2,3,4,5]. Although motors can continue operating with sparking, their performance and lifespan are significantly degraded, potentially leading to costly downtime if proper maintenance actions are not taken.

Historically, commutation quality has been measured using intrusive techniques that depend on visualizing sparks at the brush/commutator interface. These methods are not only impractical but also rely on the technician’s subjective perception and the ability to access the motor’s internal parts [6]. Advanced techniques, such as optical sensors and dipole antennas, have been proposed but face challenges like environmental interference and the need for complex installations [7,8,9,10,11,12]. Even methods based on the application of infrared thermography were proposed [13].

In recent years, vibration diagnosis [14,15] and motor current analysis have proven to be effective methodologies for diagnosing faults in DCMs. Techniques like Motor Current Signature Analysis (MCSA) have shown success in identifying increased harmonics due to sparking by analyzing the low-frequency “noise” in the current [16] and studying the current spectrum [17]. Advanced time-frequency tools, such as the Short-Time Fourier Transform (STFT), have been applied to the study of sparking activity in DCMs [2,5]. Both STFT and Wavelet Transform (WT) are used to analyze transient signals to diagnose rotor faults in induction motors [18,19,20], while the Stockwell Transform (S-Transform) has been used to calculate the Shannon entropy of a system in time-frequency diagrams, yielding good results in the mining industry [21].

This present work proposes a technique based on the acquisition and analysis of both Armature Current and Stray Axial Flux signals in transient conditions (starting transient). This study proposes two approaches to determine the temporal and frequency evolution of Shannon entropy in armature current and stray flux signals. One approach achieves this indirectly through prior analysis using the STFT, while the other directly applies the S-Transform. Experimental results show that increased sparking activity generates significant low-frequency harmonics, which are more pronounced compared to mid and high-frequency ranges, leading to a substantial rise in system entropy. This finding enables the introduction of fault-severity indicators or Key Performance Indicators (KPIs) that relate the current condition of commutation quality to a baseline established under healthy conditions. The proposed technique can be used as a predictive maintenance tool to detect and assess sparking phenomena in DCMs, providing early warnings of component failure and performance degradation, thereby enhancing the reliability and availability of these machines in both industrial environments [19], as well as electric vehicle traction systems (direct drive DC motors). Furthermore, this technique can be employed for the diagnosis of open-circuit faults in H-bridge inverters in closed-loop controlled permanent magnet synchronous motor drive systems, where the acquisition of instantaneous current samples is necessary [22].

Studies highlight the significant advantages of using combined techniques for diagnosing electric motor faults [19]. By integrating different signal analysis methods, such as monitoring both current and stray flux, the diagnostic process becomes more comprehensive and robust. This approach enhances fault detection accuracy, filters out noise, and covers a broader range of potential issues, ultimately leading to improved predictive maintenance and reduced downtime.

## 2. Materials and Methods

### 2.1. Advanced Transient Current Signature Analysis (ATCSA)

Advanced Transient Current Signature Analysis (ATCSA) represents an advanced iteration of Motor Current Signature Analysis (MCSA) [10], with a particular focus on the detection and diagnosis of faults in electrical machines, with emphasis on rotating machinery such as motors and generators. Unlike traditional MCSA, which primarily focuses on steady-state current signatures, ATCSA extends the analysis to transient-state components of the current waveform [19,20]. Transients are short-duration deviations from the steady-state current, often occurring due to events like mechanical impacts, electrical faults, or changes in load conditions. This makes it possible to detect subtle anomalies and incipient faults that may not be detected by the steady-state signature alone.

By analyzing the transient current signatures, ATCSA can detect various types of faults in electrical machines [18], including rotor bar defects [23,24], stator winding faults, bearing faults, shaft misalignment, mechanical unbalance, and commutation faults [2]. It is a sophisticated technique employed for the purposes of condition monitoring and predictive maintenance in a diverse range of industrial applications and is a non-invasive inspection tool.

Overall, ATCSA enhances the capabilities of traditional MCSA by focusing on transient current signatures and employing advanced signal-processing techniques for more accurate fault detection and diagnosis in electrical machines. It plays a vital role in ensuring the reliability, safety, and efficiency of industrial systems.

### 2.2. Stray Flux Signals Analysis

Magnetic flux analysis (MFA) has been demonstrated to be an effective alternative to conventional techniques, such as MCSA, for the condition monitoring of electric machines. Within magnetic flux analysis, we can separate between airgap flux analysis and stray flux analysis. The first is more complex and implies the installation of flux sensors in the internal parts of the machine, which is often intrusive and costly. Despite this, it has been proven to provide reliable information for the diagnosis of some faults.

On the other hand, stray flux-based methods are non-invasive and require simpler sensors. These methods imply attaching external sensors to the motor frame and monitoring the electromotive force signals that are induced in them by the stray flux. When the machine is healthy, some well-known harmonics are present in the monitored signal. Conversely, if the machine has any fault, the amplitudes of certain harmonics are increased [24,25], so these can be used to diagnose the corresponding fault. This technique has provided very satisfactory results for diagnosing certain failures in different types of electric motors, especially in induction machines, namely eccentricities, rotor problems, misalignments, or even bearing problems.

When analyzing stray flux in an induction motor, it is possible to distinguish between two principal components: the axial stray flux and the radial stray flux. Some faults manifest as distinct harmonics contingent on the portion of the flux under consideration. Consequently, an integrated analysis of both flux components may yield additional insights for diagnostic purposes [24].

**Figure 1:**
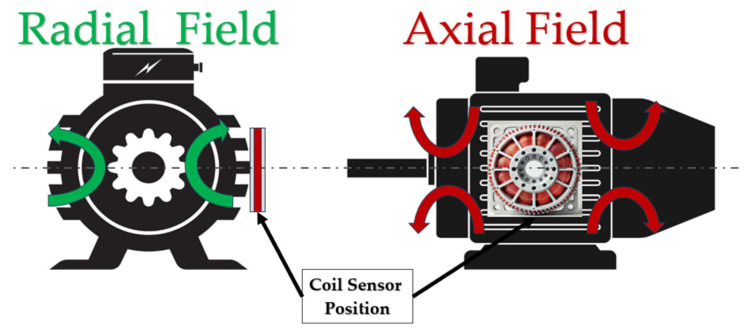
Axial and radial stray flux components [24,25,26].

The location of the coil sensor determines which portion of the flux is captured at the sensor position. Many works recommend positioning the sensor as shown in Figure 1 to simultaneously measure both portions of the flux [24,25,26]. Modern flux-based systems utilize triaxial sensors that capture the three portions of the flux at a given position by combining the data from orthogonal sub-sensors, thereby providing a comprehensive representation of the flux at that location.

### 2.3. Short-Time Fourier Transform

The Short-Time Fourier Transform (STFT) is a signal processing technique that allows for the temporal evolution of frequencies in a signal to be analyzed. The STFT segments the signal into short windows (Hanning, Hamming, or Gaussian) and applies the FFT to each segment of the signal. This allows us to examine how the frequency content of the signal changes over time, making it suitable for analyzing non-stationary signals whose frequency content varies with time.

To ensure continuity between adjacent segments and minimize spectral leakage, the windows are often overlapped with each other. After computing the Fourier transform for each segment, the resulting spectra are then overlapped and added together to reconstruct the time-frequency representation of the signal.

**Figure 2:**
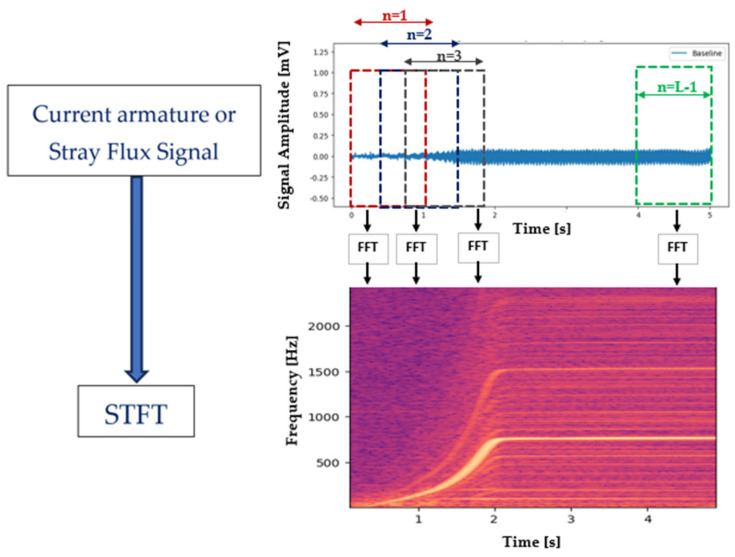
Time-frequency spectrogram resulting from the application of the STFT to the analyzed signal.

The Discrete-Time Short-Time Fourier Transform (STFT) is a version of the STFT applied to discrete-time signals. Mathematically, for a discrete-time signal $x\left[n\right]$, with length N and window of length L, the STFT at time index m and frequency bin k are computed by Equation (1).

(1) $$X_{STFT}\left[m,k\right]=\sum_{n=0}^{L-1}x\left[n+mR\right].w\left[n\right]e^{-j\frac{2\pi }{L}kn}$$

where n is the sample index within the current windows, indicating the position of the window along the signal, w[n] is the window function and R is the hop size (number of samples between successive windows, i.e., the overlap amount). Figure 2 shows in a simple way how to obtain the STFT results that can be visualized as a time-frequency spectrogram, showing the energy distribution of frequencies over time. n ranges from 0 to L−1 and represents the position of a sample within a window of length L.

The magnitude squared of the Short-Time Fourier Transform (STFT) is a commonly used representation that provides the power spectral density of a signal over time and frequency, making it useful for various applications in signal processing. The choice of window size and overlap determines the trade-off between time and frequency resolution in the STFT. Shorter windows give better time but poorer frequency resolution, and longer windows give better frequency but poorer time resolution.

### 2.4. S-Transform

The S-Transform, short for Stockwell Transform, is a time-frequency analysis technique, similar to the Continuous Wavelet Transform (CWT) with complex Morlet Wavelet [27,28], used to analyze signals in both the time and frequency domains simultaneously. It is an extension of the Short-Time Fourier Transform (STFT) and overcomes some of its limitations, such as fixed time-frequency resolution. Unlike the STFT, which has a fixed time and frequency resolution determined by the choice of window size, the S-Transform offers variable resolution using a Gaussian window. The S-Transform automatically adjusts the window length with frequency, similar to how the CWT adjusts the scale of the complex Morlet Wavelet to capture different frequency components of the signal. Both the S-Transform and the CWT provide adaptive time-frequency resolution.

This is achieved by adaptively adjusting the width of the analysis window based on the local frequency content of the signal. This allows for better resolution in both low and high-frequency regions (narrowband and broadband components) of the spectrum. However, the computational complexity of the S-Transform is higher compared to the STFT.

The S-Transform has applications in a variety of fields, including signal processing, time series analysis, geophysics [29], biomedical engineering, and audio processing. Applications include time-frequency analysis, event detection, feature extraction, and anomaly detection. From the spectrum form of S-Transform, we can derive the Discrete-Time S-Transform.

- Spectrum Form

The S-Transform definition implies that its function can be expressed as follows [29]:

(2) $$S_{x}\left(t,f\right)=\int_{-\infty }^{\infty }X\left(\tau \right)g\;\left(t-\tau ,f\right)e^{j2\pi f\tau }d\tau$$

where t is time, τ is the integration variable, f is frequency, and $g\left(t-\tau ,f\right)$ is a Gaussian window.

- Discrete-Time S-Transform of the signal at time m and frequency k can be expressed as follows [29]:

(3) Sxm,k=∑n=0N−1x[n]g n−m,ke−j2πknN

where x[n] is the discrete-time signal of discrete-time, m is the time index indicating the center of the Gaussian window as it shifts along the signal, k is the frequency index, and $g\;\left(n-m,k\right)$ is a Gaussian window, and it is inversely proportional to frequency.

### 2.5. Shannon Entropy

The Permutation Entropy (PE) statistical parameter is an indicator of the complexity or randomness of chaotic time series from the comparison of their neighboring values. It has great advantages over other parameters: speed, robustness, simplicity of calculation, and invariance to non-linear transformations. Given a time series of length N, PE first divides the series into overlapping or non-overlapping windows of a fixed length m. For each window, the ordinal pattern of the values within the window is determined. An ordinal pattern is a sequence that represents the order of the values within the window. PE computes the probability distribution of these patterns. In other words, it calculates the frequency of occurrence of each ordinal pattern within the time series.

The entropy of this probability distribution is computed using the Shannon entropy formula:

(4) $$h(m,k)=-\sum_{i=1}^{n}p_{i}{log}_{2}\left(p_{i}\right)$$

where $p_{i}\;$is the probability of the *i*-th ordinal pattern occurring and *n* is the total number of distinct ordinal patterns. The resulting entropy value provides a measure of the complexity or irregularity of the time series. Higher entropy values indicate greater complexity or randomness, while lower values indicate more ordered or predictable behavior.

In the case of analysis of the time-frequency spectrum as a result of using the STFT or S-T, entropy represents the probability of occurrence of different frequencies using the power spectral density of the spectrogram, such as:

(5) $$h(m,k)=-\sum_{m,k}\left[p_{i}\left(m,k\right)\log\left(p_{i}\left(m,k\right)\right)\right]$$

where m,k belong to the R region of the spectrum and $p_{i}\left(m,k\right)=\frac{{\left|X_{STFT}\left[m,k\right]\right|}^{2}}{\sum_{t,f}{\left|X_{STFT}\left[m,k\right]\right|}^{2}}$ represents the probability distribution of the STFT energy magnitudes at each time-frequency point.

## 3. Proposed Methodology

This section deals with the definition of the proposed methods of analysis for sparking detection in brushed DC motors/generators transient states. During the startup transient, the current and stray flux reach a value in their amplitude until they arrive at a steady state. If there is a fault in the generator, some harmonics are amplified in the spectrum of the armature current and stray flux; also, their pattern of evolution can be observed in time-frequency spectrograms. The first part describes signal acquisition, and then three methods are described. The evolution of entropy along time and frequency is shown in methods 1 and 2.

**Figure 3:**
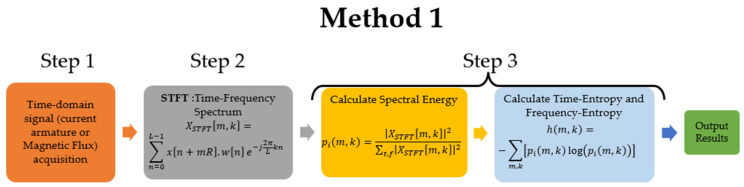

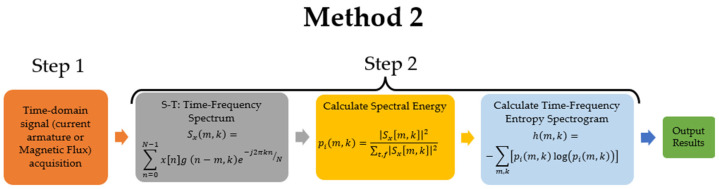
Flow chart of the methodology proposal for armature current and stray flux signals.

The proposed methodology is illustrated in Figure 3 based on the above theoretical framework.

Method 1.

- Step 1. Record armature current and axial stray flux signals simultaneously during the start-up transients + steady state using an oscilloscope, a current clamp sensor, and a magnetic flux sensor [26,30].
- Step 2. The STFT (2D or 3D) is applied by means of discrete analysis to obtain a time-frequency spectrogram of the current and stray flux signals.
- Step 3. Calculate the probability distribution of the STFT $\left(p_{i}\left(m,k\right)\right)$, then calculate Shannon entropy applying Equation (5). Shannon entropy is plotted over time and frequency.

Method 2.

- Step 1. Record armature current and axial stray flux signals simultaneously during the start-up transients + steady state using an oscilloscope, a current clamp sensor, and a magnetic flux sensor [26,30].
- Step 2. Apply Stockwell-T to obtain a time-frequency map (using Shannon entropy) of the recorded current and stray flux. Using this transformation, we can confirm the previous analyses and clearly visualize the evolution of the fault components and the higher energy frequency range.

## 4. Experimental Setup

**Figure 4:**
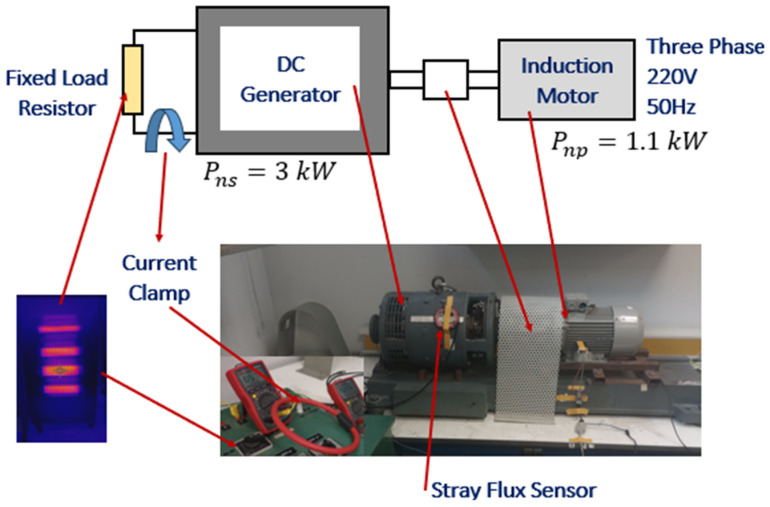
Experimental testbench: induction motor, DC generator. Measurement devices for the registration of the signals: Yokogawa DL850 waveform recorder, Fluke current clamp model CA i3000s Flex [5]. Coil sensor for measuring stray flux [24,26].

The test bench scheme is illustrated in Figure 4. The setup comprises several essential components that are employed during the tests. The comprehensive test bench has been configured for the purpose of analyzing the performance of the DC power generation system. The primary induction motor $\left(P_{np}=1.1\;kw\right)$ serves as the central component of this setup, providing the driving force for the entire system (see Table 1 for specifications). The secondary DC generator $\left(P_{ns}=3\;kw\right)$ is coupled with the induction motor and is responsible for the generation of DC power (see Table 2 for specifications). To ensure the safe dissipation of the generated energy, a resistor bank with a maximum capacity of 7.5 A is employed.

For signal acquisition, the setup employs a Yokogawa DL850 waveform recorder (specifications available on the Yokogawa website: https://tmi.yokogawa.com/es/solutions/discontinued/dl850dl850v-scopecorder/, accessed on 29 June 2024), a Fluke current clamp model CA i3000s Flex for capturing start-up current signals (specifications available on the Fluke website: https://www.fluke.com/en/product/accessories/current-clamps/fluke-i3000s-flex-24, accessed on 29 June 2024), and a specialized coil sensor for measuring stray flux (specifications in reference [24]). Furthermore, a Schneider ATS01N109FT soft starter is integrated into the system to facilitate the induction motor start-up process in an optimal manner (see Table 3 for specifications or on the Schneider website).

**Figure 5:**
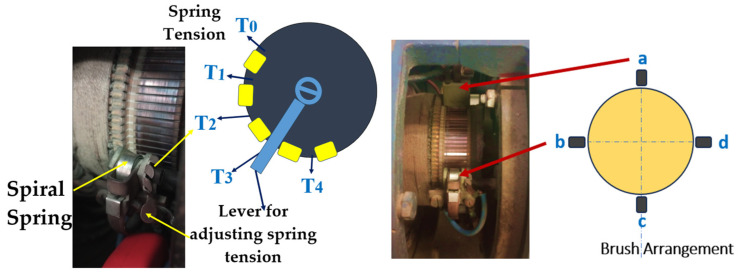
(LH) Typical configuration of the spiral spring [31], spiral spring pressure T0 (gap between brush and commutator), T1 (lower spring tension), T2, T3 (intermediate spring tension), T4 (maximum spring tension). (RH) Brush configuration, a–d represent brush positions.

The DC generator is configured with two sets of brushes for commutation, with one set distributed on either side of the generator as illustrated in Figure 5 (right). The contact pressure between the brushes and the commutator can be adjusted by varying the tension of the brush holder’s spiral spring. The spring tension can be set to four different levels: T4 represents the highest tension, T1 is the lowest, and T0 indicates no tension, meaning the brush has a slight gap with the commutator. The specific measurement and control of spring tension are beyond the scope of this paper. Figure 5 (left) depicts the spring tension positions of the brush holder.

Experimental Procedure: a total of 600 motor start-up tests were conducted (from 0 to rotational frequency), with 10 repetitions for each state at a sampling frequency of $f_{s}=10\;kHz$, and the acquisition time was set in 60 s. The tests were conducted under the following conditions: an armature current (7A, 5.65 A and 3.75 A), an excitation current (0.4 A, 0.218 A and 0.125 A), and a rotational frequency of $f_{r}=24\;Hz\;\left(1440\;rpm\right),$ $24.37\;Hz\left(1462\;rpm\right),\;$and 24.7 Hz (1482 rpm).

It is crucial to acknowledge that the rotational speed $f_{r}$ depends on the slip factor of the induction motor that serves as the primary driver. As the load increases, the slip increases, and as the load decreases, the speed approaches the synchronous rotational frequency (in this case, the optimal frequency is 25 Hz, which corresponds to 1500 rpm). Consequently, the fundamental harmonics will always be multiples of $f_{r}$. The tests encompassed a range of brush pressure levels, from T1 to T4, in order to ascertain the baseline performance of the motor when operating within healthy parameters.

**Figure 6:**
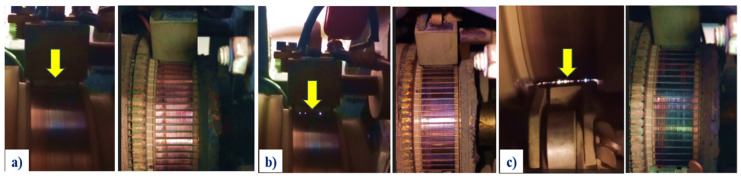
(**a**) Healthy generator (baseline), (**b**) moderate spark level, (**c**) high level of sparking activity. The yellow arrows indicate the zone of spark activity.

Furthermore, an additional 200 tests were conducted with the objective of simulating faults by inducing sparking. This was achieved by setting one brush to T0 (no tension) and combining it with tension levels T1 to T4 for the remaining three brushes, thereby analyzing nine distinct operational states. Figure 6 shows a healthy generator (Figure 6a), a moderate level (Figure 6b), and a high level of spark activity (Figure 6c). These last states are typical of a major deterioration of the brushes and the commutator surface, accompanied by an increase in temperature.

As illustrated in Figure 6, the occurrence of sparks is concentrated at the points of contact between the brushes and the commutator. Visual inspection is employed to confirm the level of brush sparks [32], the contact points are changing at every instant of time due to the change of the commutation surface.

## 5. Analysis at Steady-State

The associated harmonics are related to the rotational frequency of the induction motor ($f_{r}$) as the primary and main frequency (50 Hz). The associated harmonics are integer multiples of the rotational frequency $f_{r}$ ($2f_{r},\;3f_{r},\ldots ,\;Cf_{r},until\;C_{máx}=N_{d}$). The experimental results revealed that a harmonic with very high energy was given by $f_{p}=\frac{f_{pb}}{3}$, where $f_{pb}=N_{d}f_{r}$, also known as commutation bar passing frequency [17]. $f_{r}=24\;Hz\;(1440\;rpm)$, and commutator segments $N_{d}=96$, $f_{pb}=2304\;Hz$, and $f_{p}=32f_{r}=68\;Hz$. It is important to note that, although the selected sampling frequency is 10 kHz, which allows for the generation of a 5 kHz spectrum, the bar passing frequency $f_{pb}=2304\;Hz$ implies that the FFT should be calculated up to 2.5 kHz. This ensures the visualization of all fundamental harmonics of each state or signal analyzed, both for the armature current and the magnetic flux.

The graphs in the following sections present the time and frequency ranges that were identified by the research team as being appropriate for observing and comparing the variation of the current state signal with a baseline signal. This selection was made based on a scientific analysis of the data.

**Figure 7:**
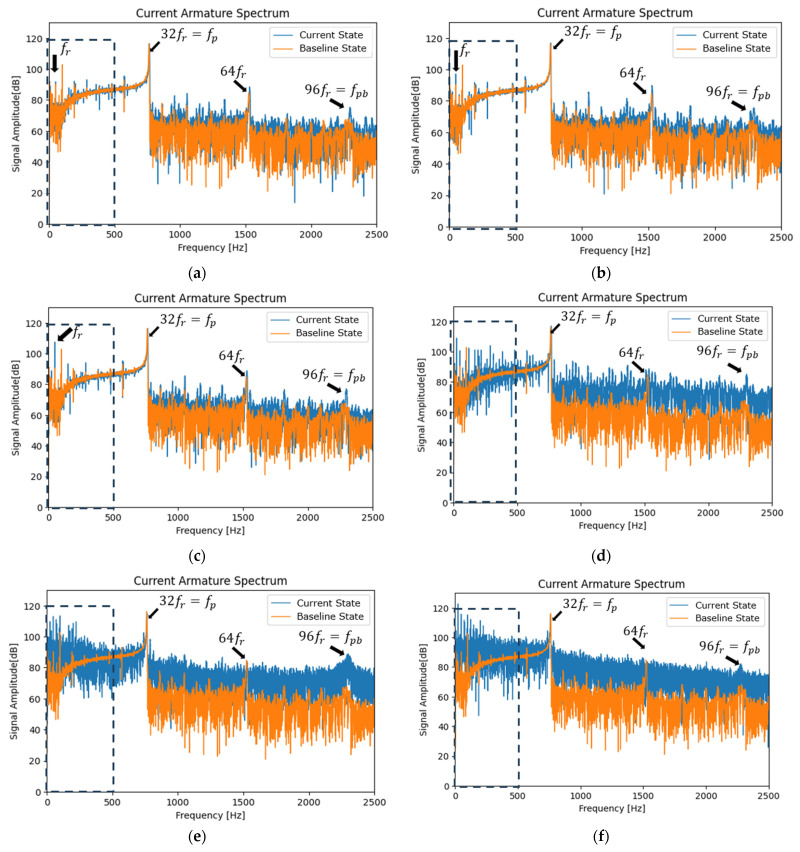
FFT spectrum (baseline vs. current state) of the armature current at a starting transient and steady state for (**a**) healthy motor, (**b**,**c**) incipient fault condition, (**d**) moderate sparking level, and (**e**,**f**) severe sparking levels.

**Figure 8:**
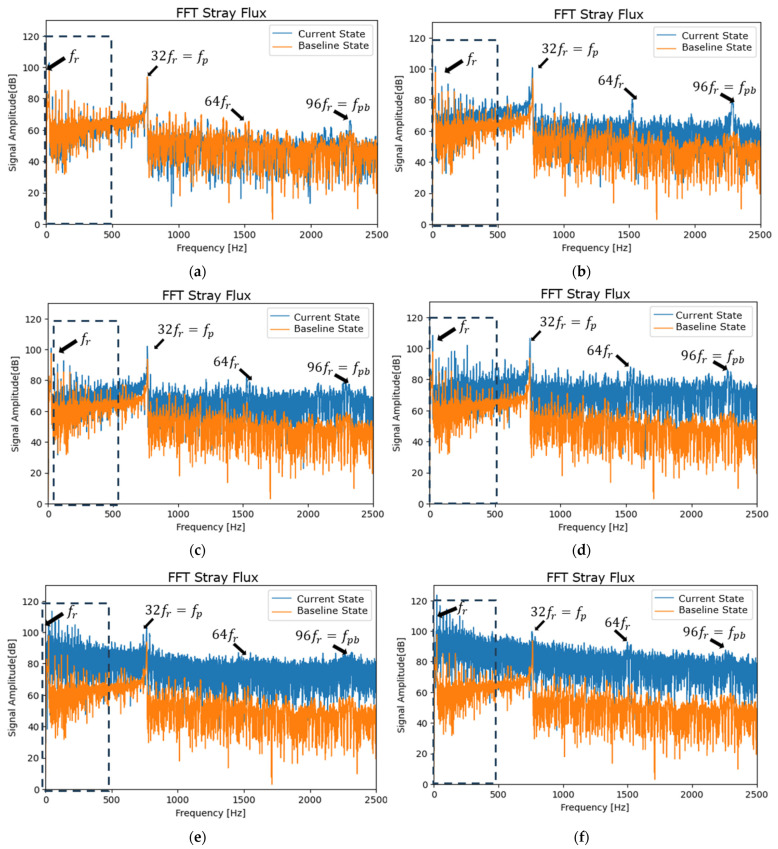
FFT spectrum (baseline vs. current state) of the stray flux at a starting transient and steady state for (**a**) healthy motor, (**b**,**c**) incipient fault condition, (**d**) moderate sparking level, and (**e**,**f**) severe sparking levels.

Figure 7 and Figure 8 show the FFT spectra of the armature current and stray flux for healthy and different sparking faults (incipient (a), (b), and (c) and severe (d), (e), and (f)) levels. The first 5 s of the signals (according to experimental procedure) have been analyzed. The first part is transient, and the rest is stationary. Note the pronounced increases in the relevant (it extends from low to high frequencies) fault harmonics, which are particularly evident for the moderate and severe fault conditions. These analyses confirm the presence of the fault components mentioned above. Their amplitudes increase progressively as the fault worsens.

In Figure 7 and Figure 8, steady-state FFT analyses show that, when spark activity occurs, low-frequency harmonics (0–500 Hz) show the highest activity compared to high frequencies. This has already been demonstrated in previous research [2,17]. The behavior is attributable to incomplete commutation.

**Figure 9:**
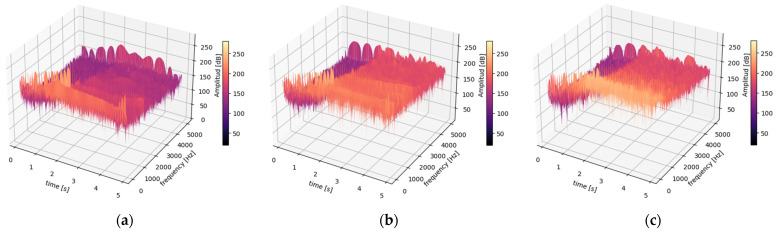
STFT 3D armature current at a transient state for (**a**) healthy motor, (**b**) moderate sparking fault condition, and (**c**) severe sparking fault condition.

**Figure 10:**
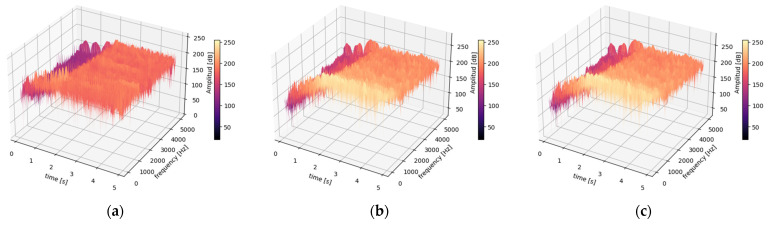
STFT 3D stray flux at a transient state for (**a**) healthy motor, (**b**) moderate sparking fault condition, and (**c**) severe sparking fault condition.

In order to gain a deeper understanding of spark activity, it is recommended to employ signal processing techniques that are more appropriate than FFT, such as STFT and S-T. These techniques facilitate a comprehensive examination of the armature current and stray flux during the start-up transient, as illustrated in Figure 9 and Figure 10.

## 6. Results and Discussion

This section analyzes the two proposed methods (see Figure 3) and describes the results obtained from the processing of signals obtained in the laboratory during the transient start-up of the machine. The evolution of the faults is illustrated in time-frequency spectrograms, (see Figure 2), and then the time-entropy and frequency-entropy diagrams are calculated.

### 6.1. Method 1

In the time-frequency analysis of a non-stationary signal, there are two conflicting requirements related to the selection of the analysis window. On one hand, the window length L must be large enough to provide the desired frequency resolution; on the other hand, it must be small enough to maintain temporal precision, allowing for the correct identification of the exact moment when each frequency evolution occurs. This duality presents a challenge in selecting the optimal window length to achieve a balance between temporal and frequency resolution. Initially, we started with a window size of L = 1024 samples and a 75% overlap, and then we increased the window size and the overlap in order to achieve the best possible resolution in both time and frequency domains. Additionally, a specific type of window and filter were carefully selected to mitigate noise and address the Nyquist effect. The subsequent sections present the results obtained from these adjustments.

Figure 9 and Figure 10 show the results obtained (Step 2 of Method 1) when calculating the STFT (3D) of the current and magnetic stray flux signals, respectively, for a healthy DC generator and a generator working under varying levels of spring pressure to the brush. The STFT was performed using a Tukey window of length L = 4096 samples and a hop size R with a 99% overlap between consecutive windows; N = 50,000 samples, $f_{s}=10\;kHz$. A bandpass filter was designed with a low-cut frequency of 5 Hz and a high-cut frequency of 4999 Hz. The filter removes frequency components outside this range. The STFT equation used is (1).

Figure 9 and Figure 10 present 3D time-frequency $\left[0-5\;s\right],\;\left[5-5000\;Hz\right]$ spectrograms that illustrate the energy changes in the spectrogram (intensity, represented by the “magma” color palette) as the generator transitions from a transient state to a steady state during start-up. The spectrograms were obtained by calculating the STFT (see Equation (1)) for each state, expressed in decibels [dB], for the armature current and magnetic flux signals under the following conditions: (a) healthy motor, (b) moderate sparking fault condition, and (c) severe sparking fault condition. A summary of the results for each case can be found below.

Thus, it is therefore evident that it is not possible to discern a clear difference (in terms of frequency content) between the healthy generator and the generator within the armature current signals when the sparking activity is considered since similar t-f maps are obtained. In contrast, if this same analysis is carried out for stray flux, it is more evident that there is a clear difference between a healthy generator and a generator with sparking when the initial conditions are the same. These findings underscore the necessity and significance of integrating the data from current signals and magnetic stray flux simultaneously.

For step 3, considering that each state (current state to every signal) resulting from applying the STFT is an energy matrix where each element of the matrix corresponds to the energy (or power) of a particular frequency in a specific time interval, mathematically it is explained if the STFT of a signal $x\left(t\right)$ is then the energy matrix ${\left|X\left(t,f\right)\right|}^{2}$, where $\left|X\left(t,f\right)\right|$ is the magnitude of the STFT coefficient. In this context, the information derived from the STFT coefficients can be employed to ascertain the Shannon entropy for each instant in time (using Equation (5) and its evolution in the transient state up to its steady-state evolution. To ensure the analysis’s relevance, we calculated the evolution of entropy for different load conditions of the generator, which are significantly lower than the standard operating requirements. This approach was taken to validate the robustness of our proposed technique and to demonstrate the sensitivity of each monitoring method. The obtained values can then be plotted as time-entropy.

**Figure 11:**
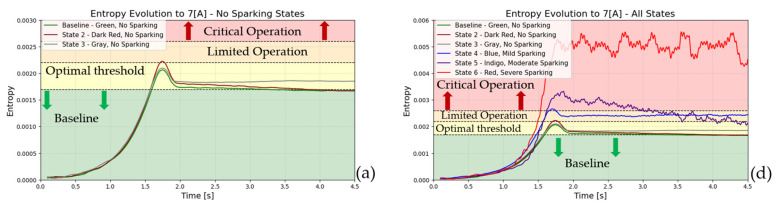

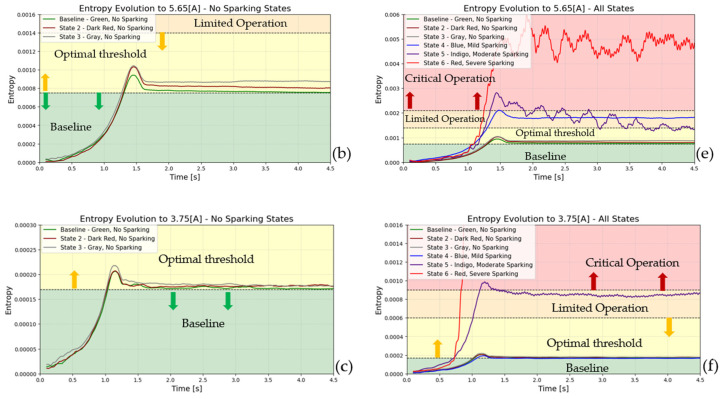
Entropy-time (2D), evolution during start-up transient for armature current signals: (**a**) to 7 [A], (**b**) to 5.65 [A], and (**c**) to 3.75 [A] represent baseline and incipient failures. (**d**) to 7 [A], (**e**) to 5.65 [A], and (**f**) to 3.75 [A] are comparative incipient failures with severe states.

**Figure 12:**
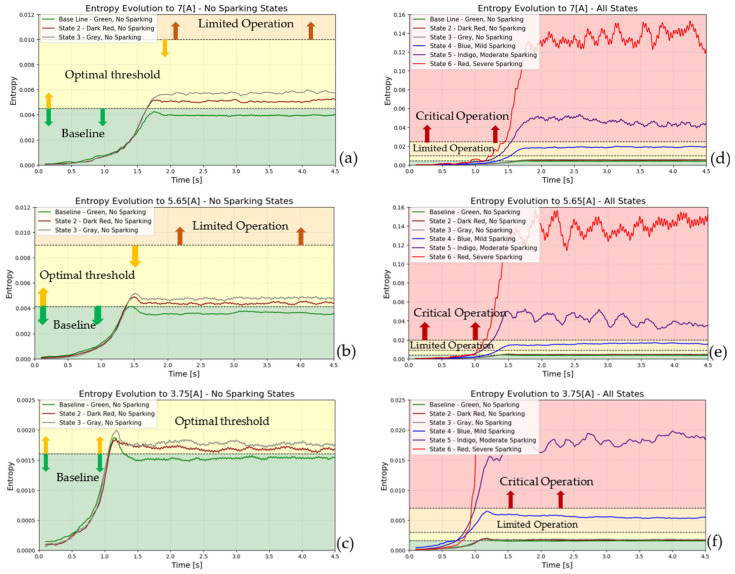
Entropy-time (2D), evolution during start-up transient for stray flux signals: (**a**) to 7 [A], (**b**) to 5.65 [A], and (**c**) to 3.75 [A] represent baseline and incipient failures. (**d**) to 7 [A], (**e**) to 5.65 [A], and (**f**) to 3.75 [A] are comparative incipient failures with severe states.

Figure 11 and Figure 12 illustrate the six states in time-entropy diagrams (armature current and stray flux signals) of a DC generator (7 A, 5.65 A,and 3.75 A of load, respectively). The initial state, designated as state 1 or the baseline (optimal operation), is followed by states 2 and 3, which exhibit signs of incipient failure (no sparking activity). Finally, states 4, 5, and 6, which represent mild, moderate, and severe levels of sparking activity, respectively, conclude this sequence.

Figure 11 and Figure 12 illustrate the entropy evolution during the start-up phase. As illustrated in Figure 12, the stray flux technique is demonstrably more sensitive than the armature current method. This sensitivity allows for the visualization of the spark phenomenon in its incipient phase, even under various partial load conditions $\left(7\;\left[A\right],\;5.65\;\left[A\right],and\;3.75\;\left[A\right],respectively\right)$. The ability to detect sparking activity is of particular importance from a diagnostic perspective when the load level is lower. Consequently, the high sensitivity of the employed technique enables the identification of even minor incipient fault conditions. As the load demand increases, the detection of such faults becomes progressively easier.

Given that the signals in question exhibit different characteristics, it is to be expected that the amplitude scales will differ between the two techniques under consideration. However, when the harmonic failure modes are compared, it is possible that they will coincide in the frequency of failure, despite their differing amplitudes.

**Figure 13:**
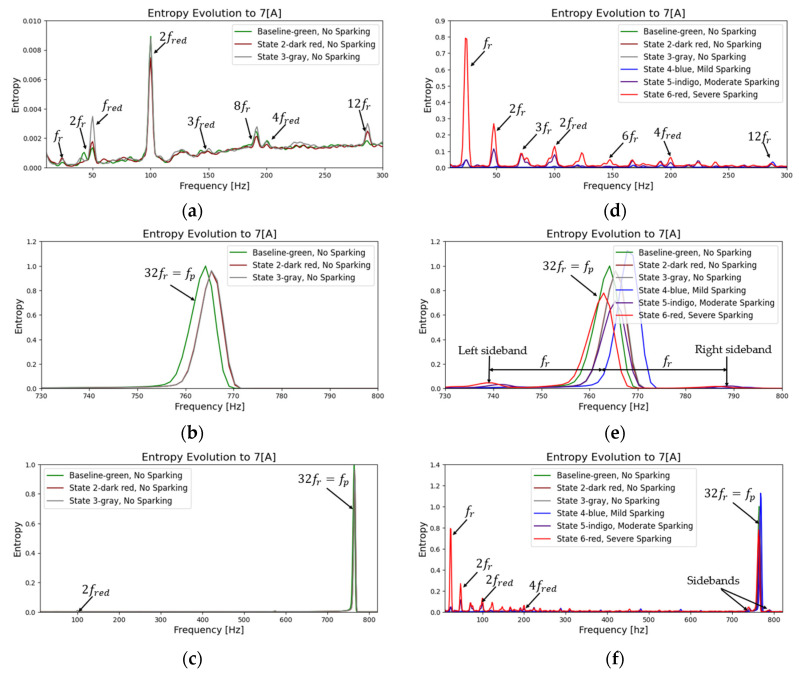
Entropy-frequency (2D), evolution during start-up transient for armature current signals. (**a**–**c**) Baseline and incipient failures, (**d**–**f**) comparative incipient failures with severe states.

**Figure 14:**
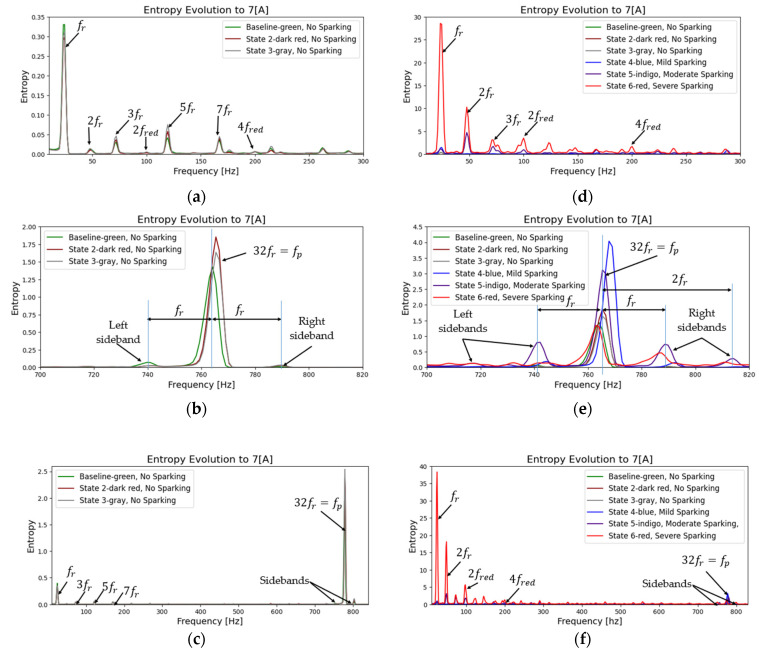
Entropy-frequency (2D), evolution during start-up transient for stray flux signals. (**a**–**c**) Baseline and incipient failures, (**d**–**f**) comparative incipient failures with severe states.

As a further step in the process, the evolution of the fault can be confirmed by repeating the same procedure and plotting a frequency-entropy diagram. This allows us to observe the activity or evolution of the fundamental harmonic rotation of the motor, as well as its multiples ($2f_{r},\;3f_{r}\;\;upto\;\;N_{d}f_{r}$) and the harmonic of greater energy ($32f_{r}=f_{p}$). Figure 13 and Figure 14 illustrate the evolution in the amplitude of sidebands in the harmonic of higher energy ($32f_{r}=f_{p}$) equidistant from the spark activity. This confirms the failure due to spark activity. It is observed that the stray flux signals are more sensitive and allow us to better define the evolution of failure and confirmation according to the time-entropy and frequency-entropy diagrams, respectively.

Figure 13a–c illustrates the change in amplitude of the $f_{red}$ (50 Hz) and its main harmonic ($f_{p}$) as the fault progresses in incipient modes. As the 50 Hz frequency decreases, the amplitude of the main harmonic increases. In contrast, in Figure 13d–f, when spark activity is present, the fundamental frequency ($f_{r}$) and its harmonics increase exponentially at low frequencies due to the incomplete commutation phenomenon. This results in a decrease in the $f_{p}$ and the emergence of sidebands at a distance from $f_{r}$, which confirms the fault.

Analyzing the results of the magnetic flux signals, Figure 14 shows how the fundamental frequency ($f_{r}$) and the main harmonic ($f_{p}$) increase in amplitude as the brush spring loses tension and the contact pressure between the brushes and the commutator decreases. In other words, we can observe the evolution of the fault in its incipient phases. In addition, low-amplitude sidebands appear at the $f_{p}$ harmonic.

On the other hand, in fault mode (see Figure 14d–f), the harmonics at low frequencies increase exponentially; both the main frequency ($f_{red}$) and $f_{r}$ increase and their multiples increase in energy. On the contrary, the main harmonic ($f_{p}$) loses energy as the fault becomes more critical, and double sidebands appear, confirming the severity of the fault.

**Figure 15:**
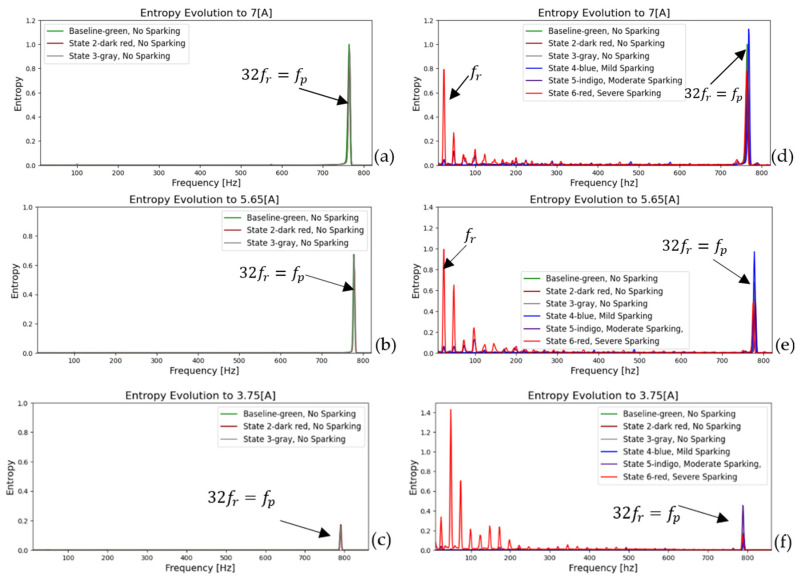
Entropy-frequency (2D), evolution during start-up transient for armature current signals: (**a**) to 7 [A], (**b**) to 5.65 [A], and (**c**) to 3.75 [A] represent baseline and incipient failures. (**d**) to 7 [A], (**e**) to 5.65 [A], and (**f**) to 3.75 [A] are comparative incipient failures with severe states.

**Figure 16:**
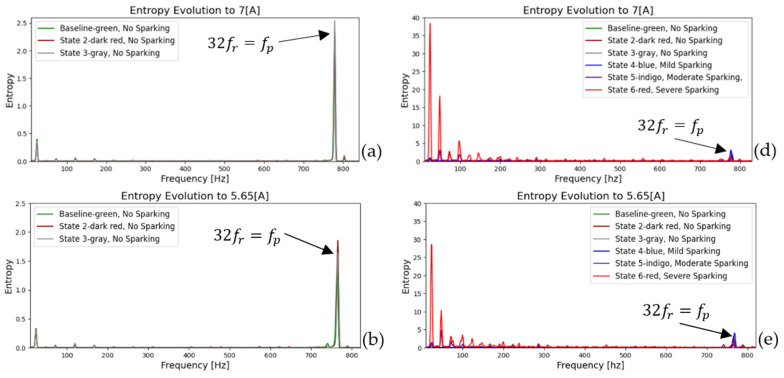

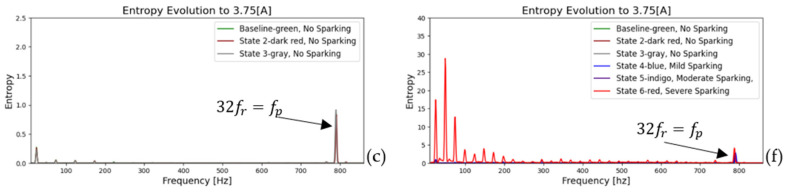
Entropy-frequency (2D), evolution during start-up transient for stray flux signals: (**a**) to 7 [A], (**b**) to 5.65 [A], and (**c**) to 3.75 [A] represent baseline and incipient failures. (**d**) to 7 [A], (**e**) to 5.65 [A], and (**f**) to 3.75 [A] are comparative incipient failures with severe states.

To complete our analysis, we calculated the frequency evolution of entropy for different load levels. This allows us to further demonstrate the robustness of the method and to visualize the sensitivity of each monitoring technique. Figure 15 and Figure 16 illustrate the evolution of the magnitude of low-frequency harmonics and the main harmonic as the load decreases for both the armature current technique and the magnetic flux technique. It is evident that the harmonics are practically identical in both cases, although at lower loads, the amplitude of these harmonics is reduced.

### 6.2. Method 2

In order to confirm the low-frequency energy activity and the main harmonic energy activity due to the defect associated with spark activity in DC motors/generators, a direct method of entropy analysis in time-frequency maps using the Stockwell transform is studied next. The change in energy distribution can be calculated using energy entropy. The entropy will change as the energy distribution changes. Traditional energy entropy, however, cannot detect both the time and location of changes because it is calculated only in the time or frequency domain (Method 1).

Time-frequency-entropy measures any distribution of information using time and frequency information and is widely used in many practical applications, such as hydrocarbon extraction systems [21] and machinery fault diagnosing [33]. In this regard, in order to avoid the high computational complexity for classification, some authors proposed S-Transform (S-T) based on STFT with Gaussian windowing, which will be applied in this section.

In addition, new time-frequency-entropy methods are an evolution of S-T, such as the generalized S-Transform (GST), synchro-squeezing generalized S-Transform (SSGST), and the so-called high precision due to focus on the instantaneous frequency of the signal [21].

In accordance with the methodology detailed for Method 2 in Figure 3, this approach is based on a direct analysis to jointly obtain the evolution of Shannon entropy (Equation (5)). This is achieved by first applying the S-Transform (Equation (3)) with a window size of 1024 samples and a 50% overlap of the Gaussian window. In addition to the states previously analyzed in the preceding section, three more states in the incipient fault stage have been included to further demonstrate the effectiveness and interest of the method.

**Figure 17:**
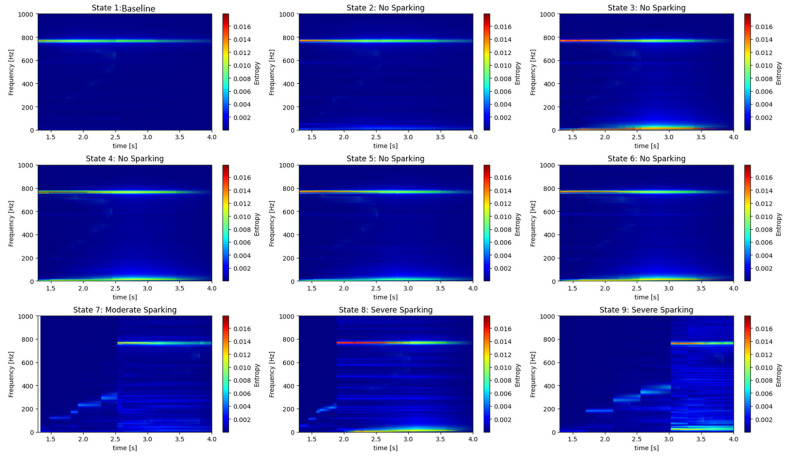
S-T, time-frequency maps obtained by analyzing the armature current signals of the DC generator for different start-up transients to 7 [A]. States 1, 2: healthy generator; states 3, 4, 5, and 6: incipient failure; states 7, 8, and 9: moderate and severe sparking activity.

**Figure 18:**
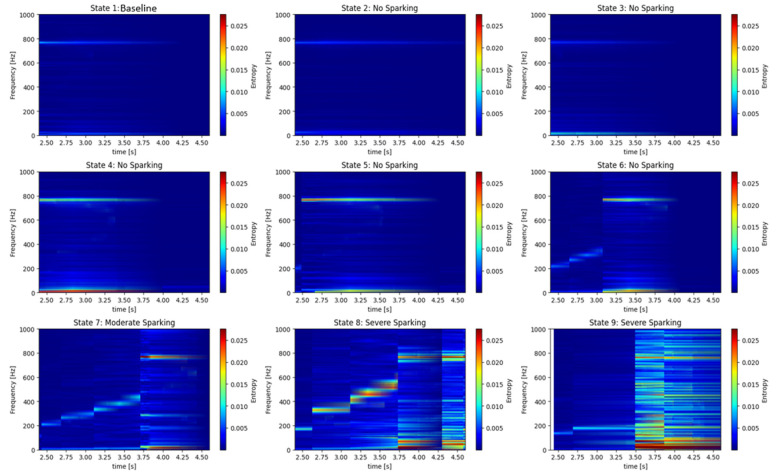
S-T time-frequency maps obtained by analyzing the stray flux signals of the DC generator for different start-up transients to 7 [A]. States 1, 2: healthy generator; states 3, 4, 5, and 6: incipient failure; states 7, 8, and 9: moderate and severe sparking activity.

Figure 17 and Figure 18 illustrate the time-frequency maps of entropy during the start-up phase to 7 [A]. In particular, states 1 and 2 represent the *baseline*, or healthy generator, while states 3, 4, 5, and 6 indicate incipient failures, characterized by the absence of sparking. States 7, 8, and 9 correspond to moderate and severe failures, as evidenced by the presence of sparking activity. Figure 17 and Figure 18 illustrate the evolution of the harmonics of higher energy of the armature current and stray flux signals. When the brush spring tension is lost, when brush wear increases, or when the sparking phenomenon appears, the S-Transform locates the zone of highest activity frequencies. However, it is important to distinguish between a fundamental harmonic and a fault.

Armature Current

Stray Flux

A review of the S-Transform graphs applied to both armature current and stray flux signals under different operating states of the generator reveals the following conclusions:

The optimal operating states, as indicated by states 1 and 2, are characterized by the following: The graphs corresponding to states 1 and 2 demonstrate a clear and stable frequency spectrum, with minimal activity in the low-frequency range. This suggests that the generator is operating optimally, without the presence of faults.

Incipient fault states (states 3, 4, 5, and 6): In these states, an increase in activity in the low frequencies of the spectrum is observed. The presence of these low frequencies is indicative of incomplete commutation and the emergence of issues in the generator’s brush system. The S-Transform enables the identification of these nascent faults prior to their escalation into significant issues, thereby offering a valuable instrument for predictive maintenance.

Fault states (States 7, 8, and 9): In these states, considerable activity is discernible in the spectrum, particularly in the low and mid frequencies. The amplitude of these frequencies is markedly elevated in comparison to the optimal and incipient states, indicative of the presence of severe faults.

The severity of the faults increases progressively from state 7 to state 9, with a more complex spectrum and multiple harmonics in the low and mid frequencies. This suggests that the generator is experiencing severe problems, potentially related to incomplete commutation and loss of tension in the brush springs.

Method 1 offers significant benefits by enabling early fault detection through the generation of detailed time-entropy and frequency-entropy maps. These maps allow for the identification of the evolution of main harmonics associated with sparking faults, facilitating the establishment of reference thresholds for healthy, alert, and critical conditions of the motor/generator. This non-intrusive approach relies on the analysis of armature current and stray flux signals, thus enhancing safety and feasibility in various industrial settings. However, the complexity of signal processing involved in this method requires advanced knowledge of Short-Time Fourier Transform (STFT) and Shannon entropy (PE), along with specialized computational resources. Additionally, ensuring high-quality signal acquisition is essential for accurate analysis, which might necessitate precise and potentially costly instrumentation.

Method 2 provides a comprehensive evaluation of sparking activity by integrating information from both current and stray flux signals, thereby enhancing the accuracy of fault detection and offering a broader understanding of the motor/generator’s health. The utilization of the S-Transform for time-frequency-entropy analysis provides superior resolution and adaptability, rendering it an effective methodology for the detection of transient and non-stationary faults. The capacity of this method to furnish detailed insights into the root causes of sparking faults renders it an invaluable tool for predictive maintenance. However, the complexity of simultaneously analyzing multiple signals and applying the S-Transform increases the implementation challenges, necessitating the use of advanced software tools and expertise. Furthermore, the computational load associated with the integration process and entropy calculation can be considerable, potentially limiting the feasibility of real-time applications.

These methods are complementary, and their integration can provide a robust framework for monitoring and maintaining the health of DC motors/generators in industrial applications.

## 7. Conclusions

This paper presents two innovative non-intrusive methods for the evaluation of sparking activity in DC motors/generators, employing Shannon entropy for the early monitoring of sparking activity. The methods are based on the steady-state and transient analyses of armature current and stray flux signals, with a particular focus on those acquired during the system start-up phase.

The initial method entails the processing of signals to generate time-entropy and frequency-entropy maps (applying STFT and PE), thereby facilitating the identification of the evolution of principal harmonics associated with sparking faults. This approach also enables the definition of thresholds for a healthy motor/generator, a motor/generator with a fault alert, and a motor/generator in critical condition. Consequently, reference thresholds can be established, and appropriate maintenance actions can be adopted.

The application of the S-Transform to both armature current and stray flux signals has proven to be an effective tool for the detection and diagnosis of faults in electrical generators. The ability of this technique to provide a detailed time-frequency representation allows for the identification of subtle changes in the spectrum, facilitating the early detection of incipient faults and the assessment of their severity. This is crucial for implementing predictive maintenance strategies and avoiding catastrophic failures in generators.

These two methods are mutually reinforcing. By integrating the time-entropy and frequency-entropy maps with the combined analysis of current and stray flux signals, a more comprehensive and accurate evaluation of the sparking activity and overall health of the motor/generator can be achieved. Finally, these methods can be automated for industrial applications.

## Figures and Tables

**Table 1:** Rated values and characteristics of induction motor (primary).

Power (kW)	1.1
Main Frequency (Hz)	50
Voltage (V)	400
Current (A)	2.4
Speed (rpm)	1440
Connection	Star
Number of Pole Pairs	2

**Table 2:** Rated values and characteristics of the Bruh DC machine (secondary).

Power (kW)	3
Main Frequency (Hz)	50
Voltage (V)	220
Excitation Current (A)	0.4
Armature Current (A)	13.6
Speed (rpm)	2000
Commutator Segments ($N_{d}$)	96

**Table 3:** Characteristics of the industrial soft starter used during tests. Product website: https://www.se.com/us/en/product/ATS01N109FT/soft-starter-for-asynchronous-motor-altistart-01-ats01-9a-110-to-480v-1-1-to-4kw/ (accessed on 29 June 2024).

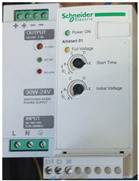	**Manufacturer**	**Schneider (Germany)**
	Model	ATS01N109FT
	Main Frequency (Hz)	50
	Voltage (V)	400
	Power(kW)	4
	Max. Current(A)	9.0
	Phases Control	1
	Voltage ramp (s)	1–5

## Data Availability

Dataset available on request from the authors.

## Abbreviations

The following abbreviations are used in this paper:

DC	Direct Current
GST	Generalized S-transform.
PE	Permutation entropy
S-T	Stockwell Transform
SSGST	Squeezing Generalized S-transform.
STFT	Short-Time Fourier Transform
